# Characterization of *BRCA1* and *BRCA2* variants in multi-ethnic Asian cohort from a Malaysian case-control study

**DOI:** 10.1186/s12885-017-3099-6

**Published:** 2017-02-22

**Authors:** Kah Nyin Lai, Weang Kee Ho, In Nee Kang, Peter Choon Eng Kang, Sze Yee Phuah, Shivaani Mariapun, Cheng-Har Yip, Nur Aishah Mohd Taib, Soo-Hwang Teo

**Affiliations:** 1 Cancer Research Malaysia, 1 Jalan SS12/1A, 47500 Subang Jaya, Selangor Malaysia; 2 Department of Applied Mathematics, Faculty of Engineering, The University of Nottingham Malaysia Campus, 43500 Semenyih, Selangor Malaysia; 3 Subang Jaya Medical Centre, 1 Jalan SS12/1A, 47500 Subang Jaya, Selangor Malaysia; 4 Breast Cancer Research Unit, University Malaya Cancer Research Institute, Faculty of Medicine, University Malaya Medical Centre, University Malaya, 50603 Kuala Lumpur, Malaysia; 5 Department of Surgery, Faculty of Medicine, University Malaya Medical Centre, 50603 Kuala Lumpur, Malaysia

**Keywords:** *BRCA1*, *BRCA2*, Variant of uncertain significance, Malaysia, Asia

## Abstract

**Background:**

Genetic testing for *BRCA1* and *BRCA2* has led to the accurate identification of individuals at higher risk of cancer and the development of new therapies. Approximately 10-20% of the genetic testing for *BRCA1* and *BRCA2* leads to the identification of variants of uncertain significance (VUS), with higher proportions in Asians. We investigated the functional significance of 7 *BRCA1* and 25 *BRCA2* variants in a multi-ethnic Asian cohort using a case-control approach.

**Methods:**

The MassARRAY genotyping was conducted in 1,394 Chinese, 406 Malay and 310 Indian breast cancer cases and 1,071 Chinese, 167 Malay and 255 Indian healthy controls. The association of individual variant with breast cancer risk was analyzed using logistic regression model adjusted for ethnicity, age and family history.

**Results:**

Our study confirmed *BRCA2* p.Ile3412Val is presented in >2% of unaffected women and is likely benign, and *BRCA2* p.Ala1996Thr which is predicted to be likely pathogenic by *in-silico* models is presented in 2% of healthy Indian women suggesting that it may not be associated with breast cancer risk. Single-variant analysis suggests that *BRCA1* p.Arg762Ser may be associated with breast cancer risk (OR = 7.4; 95% CI, 0.9–62.3; *p* = 0.06).

**Conclusions:**

Our study shows that *BRCA2* p.Ile3412Val and p.Ala1996Thr are likely benign and highlights the need for population-specific studies to determine the likely functional significance of population-specific variants. Our study also suggests that *BRCA1* p.Arg762Ser may be associated with increased risk of breast cancer but other methods or larger studies are required to determine a more precise estimate of breast cancer risk.

**Electronic supplementary material:**

The online version of this article (doi:10.1186/s12885-017-3099-6) contains supplementary material, which is available to authorized users.

## Background

Germline mutations in *BRCA1* (MIM 113705) and *BRCA2* (MIM 600185) are associated with increased risk of breast and ovarian cancer. The discovery of germline mutations has led to the accurate identification of individuals who are at risk of cancer and the development of new therapies for the disease. In many countries, individuals with *a priori* risk of 10–20% of having inherited a germline mutation in *BRCA1* and *BRCA2* are offered genetic counselling and testing [1] and on average, 17–20% of the genetic testing for *BRCA1* and *BRCA2* detect pathogenic mutations [2, 3]. However, approximately 10–20% of the tests lead to the identification of variants of uncertain significance (VUS) which comprise missense variants, intronic variants, synonymous variants and in-frame insertions or deletions [4, 5], for which the clinical relevance remains equivocal.

The frequency of VUS varies by ancestry around the world with lower frequency in populations that are well studied such as the Caucasian population in North America and Europe, and high frequency in populations such as Asian, African and Middle Eastern where there has been little study and limited availability of genetic counselling and testing [6]. Over time, VUS are systematically reclassified as additional information or evidence is supported [7, 8]. According to a study reported by Myriad Genetics in United States based on 20 years of experience, the frequency of VUS in *BRCA1* and *BRCA2* declined from 12.8 to 2.1% [6]. Notably, the frequency of VUS remains the highest in Asians with 7% of the tests reported as VUS. Although the pathogenicity of VUS has been studied using different approaches such as multifactorial likelihood model, population frequency, functional or mRNA splicing assay, the majority of these are focused on VUS in the Caucasian population [9–11]. In Asia, studies have reported novel variants but the clinical significance of these variants has not been further investigated [12–15].

In this study, we analyzed the frequency of 7 *BRCA1* and 25 *BRCA2* variants in an Asian cohort of 2,110 breast cancer patients and 1,493 healthy women, in which the pathogenicity of variants is evaluated using a case-control approach.

## Methods

### Study description

The recruitment of breast cancer patients into the Malaysian Breast Cancer Genetic Study (MyBrCa) started in January 2003 at University Malaya Medical Centre, and in September 2012 at Subang Jaya Medical Centre in Kuala Lumpur, Malaysia. All were histopathology proven breast carcinoma. Blood, demographic and family history data were collected from breast cancer patients who consented to participate in this study.

From January 2003 to March 2014, 2,323 breast cancer patients were recruited into the study. A total of 467 individuals were selected for germline analysis on the basis of age of onset and family history of breast and/or ovarian cancer, in which 402 of the cases have been previously described [15–18] and 65 additional cases were tested using the same selection criteria. Detection of germline mutations was conducted using direct DNA sequencing and multiplex ligation-dependent probe amplification (MLPA) on the *BRCA1* and *BRCA2* genes.

Of the 2,323 breast cancer patients, cases were excluded if they were of mixed parentage or ethnicities other than Chinese, Malay or Indian (*n* = 87), or had insufficient or low quality genomic DNA (*n* = 126), leaving a cohort of 2,110 individuals (1,394 Chinese, 406 Malay and 310 Indian) for genotyping. Controls were selected from 1,530 women with no personal history of breast cancer attending an opportunistic mammography screening program from October 2011 to April 2013 [19]. A total of 37 controls who were of mixed parentage or ethnicities other than Chinese, Malay or Indian (*n* = 37) were excluded. The remaining 1,493 individuals consisting of 1,071 Chinese, 167 Malay and 255 Indian with sufficient genomic DNA were genotyped.

### Selection and genotyping of variants

The germline analysis identified 69 missense and intronic variants of *BRCA1* and *BRCA2* from 109 individuals. All variants identified from the germline analysis, regardless of their predicted clinical importance, were selected for genotyping to evaluate their frequency using a case-control approach. The variants were annotated according to Human Genome Variation Society (HGVS) recommendations based on transcript sequence of *BRCA1* (NM_007294.3) and *BRCA2* (NM_000059.3). Two variants (*BRCA1* p.Asp345Tyr and *BRCA2* c.632-10dupT) failed in assay design due to repetitive nucleotides located at neighboring sequence of each variant. Of the remaining variants, 23 *BRCA1* and 44 *BRCA2* variants were included in the genotyping assay (Additional file 1: Table S1a and S1b).

Genotyping was conducted using SEQUENOM iPlex multiplex single-base extension assays and analyzed by MALDI-TOF mass spectrometry (SEQUENOM Inc., San Diego, USA). Individuals who were previously analyzed by direct DNA sequencing and MLPA were used as positive and negative controls for genotyping. The genotyping process involved two phases in which the assay of Phase 1 included 27 variants (9 *BRCA1* and 18 *BRCA2*) and tested on 879 breast cancer cases recruited from January 2003 to July 2010. In Phase 2, the remaining 40 variants (14 *BRCA1* and 26 *BRCA2*) were added to the assay and this was tested on a non-overlapping cohort of 1,231 breast cancer cases and 1,493 healthy controls recruited from July 2010 to March 2014 (Additional file 1: Table S1a and S1b). Where possible, all cases and controls participating in the research study were included in the genotyping assay. The subsequent variant analyses were performed according to the number of genotyped cases and controls for each variant. Variants with genotyping call rate of <95% were excluded. Approximately 5% of the randomly selected samples were duplicated in the experiment and samples that were failed to be genotyped in >20% of the assays were excluded.

### *In-silico* prediction

The effect of missense variants on protein function was predicted using AGVGD (http://agvgd.hci.utah.edu/), PolyPhen-2 (http://genetics.bwh.harvard.edu/pph2/) and SIFT (http://sift.jcvi.org/). AGVGD is an evolutionary sequence conservation model in which the algorithm evaluates the physiochemical properties of amino acid and multiple sequence alignments in a substituted protein sequence [20]. PolyPhen-2 uses sequence-based and structure-based predictive features to evaluate the damaging effects of missense variants [21]. SIFT predicts the effects of all possible substitutions at each position in the protein sequence by using sequence homology [22].

### Statistical analyses

The information on ethnicity, age of diagnosis or consent, and family history of breast or ovarian cancer in first and second degree relatives were obtained from the questionnaires. The association between breast cancer risk and these baseline characteristics was investigated using t-test for age and Chi-Square for ethnicity and family history. We assessed the relationship between each variant and the risk of breast cancer using logistic regression model adjusted for ethnicity, age and family history. All statistical analyses for single-variant association testing were performed using Statistical Package and Service Solutions (SPSS) version 16.0. The R package ‘rmeta’ (version 2.16; https://cran.r-project.org/web/packages/rmeta/index.html) was used to generate the forest plot of single-variant association in the Additional file 2.

## Results

We determined the spectrum of *BRCA1* and *BRCA2* deleterious mutations and variants in 467 breast cancer patients by full sequence analysis and large genomic rearrangement analysis. Of these, 69 (14.8%) had germline deleterious mutations and 125 (26.8%) had variants in *BRCA1* and *BRCA2* genes. In total, 24 *BRCA1* and 45 *BRCA2* missense and intronic variants were identified from 109 individuals. Of these 69 variants, 67 variants that could be designed for the MassARRAY platform were included in a multiplex genotyping assay (Additional file 1: Table S1a and S1b).

The genotyping assay was tested on 2,110 breast cancer cases and 1,493 healthy controls (Table 1). Majority of the individuals recruited for this study were Chinese (66.1% in cases and 71.7% in controls), followed by Malay (19.2% in cases and 11.2% in controls) and Indian (14.7% in cases and 17.1% in controls). The average age of breast cancer cases (49.5 years) was slightly younger than healthy controls (50.3 years). Notably, there was no difference in age for cases and controls for Chinese and Indian women, but healthy women were on average 2 years older than the cases for Malay women (Additional file 1: Table S2). Ethnicity, age and family history of breast or ovarian cancer were significantly associated with breast cancer risk and hence were included as covariates in single-variant association testing.

**Table 1:** Characteristics of Malaysian breast cancer cases and healthy controls

Characteristics	Breast cancer cases		Healthy controls		*p-*value
	*N* = 2,110		*N* = 1,493		
	N	%	N	%	
Ethnicity					<0.001
Chinese	1,394	66.1	1,071	71.7	
Malay	406	19.2	167	11.2	
Indian	310	14.7	255	17.1	
Age^a^ (years)					
Average age	49.5		50.3		0.009
≤ 30	76	3.6	0	0	
31–40	337	16.0	72	4.8	
41–50	751	35.6	781	52.3	
51–60	623	29.5	501	33.6	
≥ 61	323	15.3	139	9.3	
Family history^b^					0.001
Yes	506	24.0	288	19.3	
No	1,598	75.7	1,205	80.7	
No data	6	0.3	0	0	
Pathology profile					
ER+	1,218	57.7			
ER-	451	21.4			
Triple negative	242	11.5			
No data	199	9.4			

^a^ Age of diagnosis for breast cancer cases or age of consent for healthy controls

^b^ Family history of breast or ovarian cancer in first or second degree relatives

Genotyping of 2 *BRCA1* (c.-19-3A > G and c.-19-10 T > C) and 2 *BRCA2* (p.His523Arg and p.Arg2502His) variants resulted in genotyping call rates of <95%, and these variants were therefore excluded from the analysis. We also excluded 19 cases and 29 controls from analysis because these samples were failed to be genotyped in >20% of the assays. As a result, 2,091 breast cancer patients and 1,464 healthy controls were analyzed using a case-control approach. The genotyping cohort also included 120 individuals who were previously analyzed by germline analysis. Of these, genotyping was concordant with sequencing in 119 of 120 individuals (99.2%). Approximately 5% of randomly selected samples (88 cases and 67 controls) were duplicated in the genotyping assay and the concordance rate among the duplicated samples was 98.7% (153/155).

We identified *BRCA2* p.Ile3412Val with variant frequency >2% in unaffected women and an additional four variants (*BRCA2* p.Cys315Ser, p.Ile1929Val, p.Arg2108Cys and p.Lys2729Asn) with variant frequency >1% in unaffected women (Table 3). All of these are unlikely to be associated with increased risk of breast cancer.

Of the 63 variants that were included in the analysis, thirty-one variants (14 *BRCA1* and 17 *BRCA2*) could not be evaluated as carriers were present only in either breast cancer cases or healthy controls. Only 32 variants (7 *BRCA1* and 25 *BRCA2*) were present in both cases and controls and these were analyzed for association with breast cancer risk (Tables 2 and 3).

**Table 2:** *BRCA1* variants detected in Malaysian carriers by genotyping

HGVS cDNA	HGVS protein	Reported in BIC^a^	AGVGD^b^	PolyPhen-2^c^	SIFT^d^	MAF^e^ (%)	VF^f^ in controls (%)	Breast cancer cases	Healthy controls	OR^g^	95% CI	*p*-value
c.571G > A	p.Val191Ile	10	C0	Benign	Damaging	0.30	0.75	5/1218	11/1464	0.53	0.18–1.54	0.242
c.823G > A	p.Gly275Ser	4	C0	Probably damaging	Damaging	0.08	0.14	4/2089	2/1464	1.49	0.27–8.25	0.650
c.1036C > T	p.Pro346Ser	4	C0	Benign	Tolerated	0.13	0.14	5/1219	2/1464	3.30	0.64–17.09	0.155
c.2286A > T	p.Arg762Ser	3	C0	Benign	Damaging	0.13	0.07	6/1218	1/1463	7.44	0.89–62.32	0.064
c.2726A > T	p.Asn909Ile	2	C0	Possibly damaging	Damaging	0.13	0.27	5/2091	4/1462	0.90	0.24–3.37	0.874
c.3625 T > G	p.Leu1209Val	1	C0	Possibly damaging	Damaging	0.04	0.14	1/2058	2/1461	0.18	0.02–2.05	0.168
c.3662A > C	p.Glu1221Ala	3	C0	Probably damaging	Damaging	0.07	0.21	1/1215	3/1454	0.44	0.05–4.19	0.471

^a^ Breast Cancer Information Core (http://research.nhgri.nih.gov/bic/), an open access online breast cancer mutation database assessed by Oct 2015

^b^ AGVGD grades: C0, C15, C25, C35, C45, C55, C65 with C65 most likely to interfere with function and C0 least likely. The probability was assessed using the alignment of which from human to sea urchin in *BRCA1*

^c^ PolyPhen-2 grades: Benign, Possibly damaging, Probably damaging

^d^ SIFT grades: Tolerated, Damaging

^e^ Minor allele frequency, which was detected in the entire cohort

^f^ Variant frequency, which was detected only in healthy controls

^g^ Odds ratio, adjusted for ethnicity, age and family history using logistic regression

**Table 3:** *BRCA2* variants detected in Malaysian carriers by genotyping

HGVS cDNA	HGVS protein	Reported in BIC^a^	AGVGD^b^	PolyPhen-2^c^	SIFT^d^	MAF^e^ (%)	VF^f^ in controls (%)	Breast cancer cases	Healthy controls	OR^g^	95% CI	*p*-value
c.215A > G	p.Asn72Ser	1	C0	Benign	Tolerated	0.07	0.21	1/1215	3/1454	0.41	0.04–3.98	0.441
c.440A > G	p.Gln147Arg	16	C0	Benign	Tolerated	0.39	0.62	12/1218	9/1463	1.38	0.58–3.33	0.469
c.943 T > A	p.Cys315Ser	15	C0	Benign	Tolerated	0.50	1.20	18/2055	17/1420	0.79	0.41–1.55	0.496
c.1825C > G	p.Gln609Glu	1	C0	Benign	Damaging	0.06	0.07	2/1218	1/1464	1.65	0.15–18.37	0.683
c.2186 T > C	p.Ile729Thr	0	C0	Benign	Tolerated	0.09	0.28	1/1213	4/1423	0.31	0.04–2.80	0.298
c.3445A > G	p.Met1149Val	8	C0	Benign	Tolerated	0.26	0.48	7/1215	7/1454	0.83	0.29–2.43	0.740
c.4376A > G	p.Asn1459Ser	1	C0	Benign	Tolerated	0.10	0.14	5/2087	2/1462	1.89	0.37–9.80	0.448
c.4779A > C	p.Glu1593Asp	3	C0	Benign	Tolerated	0.14	0.27	6/2088	4/1464	1.15	0.32–4.13	0.834
c.5167A > C	p.Thr1723Pro	1	C0	Possibly damaging	Tolerated	0.04	0.07	1/1214	1/1454	1.05	0.06–16.99	0.976
c.5312G > A	p.Gly1771Asp	44	C0	Benign	Tolerated	0.03	0.07	1/2054	1/1459	0.58	0.04–9.74	0.706
c.5785A > G	p.Ile1929Val	32	C0	Benign	Tolerated	0.82	1.62	20/1213	23/1421	1.03	0.56–1.90	0.916
c.5986G > A	p.Ala1996Thr	4	C55	Probably damaging	Tolerated	0.16	0.34	6/2060	5/1462	0.95	0.29–3.16	0.932
c.6322C > T	p.Arg2108Cys	22	C0	Benign	Tolerated	0.55	1.12	13/1207	16/1431	0.94	0.45–1.98	0.871
c.6325G > A	p.Val2109Ile	8	C0	Benign	Tolerated	0.25	0.49	6/1213	7/1422	0.89	0.30–2.69	0.837
c.7052C > G	p.Ala2351Gly	6	C0	Possibly damaging	Damaging	0.21	0.49	8/2077	7/1422	0.78	0.28–2.17	0.627
c.7469 T > C	p.Ile2490Thr	240	C0	Benign	Tolerated	0.06	0.07	3/2060	1/1462	2.27	0.24–21.90	0.480
c.8187G > T	p.Lys2729Asn	24	C0	Probably damaging	Damaging	0.63	1.30	15/1218	19/1463	0.98	0.50–1.95	0.964
c.8356G > A	p.Ala2786Thr	2	C0	Probably damaging	Damaging	0.17	0.28	8/2088	4/1448	1.52	0.45–5.07	0.498
c.8702G > A	p.Gly2901Asp	3	C65	Probably damaging	Damaging	0.11	0.34	1/1217	5/1464	0.25	0.03–2.16	0.208
c.9104A > G	p.Tyr3035Cys	3	C55	Probably damaging	Damaging	0.06	0.07	2/1213	1/1454	2.34	0.21–26.02	0.489
c.9538C > T	p.Leu3180Phe	1	C0	Probably damaging	Damaging	0.04	0.14	1/2089	2/1448	0.34	0.03–3.81	0.382
c.10234A > G	p.Ile3412Val	114	C0	Benign	Tolerated	1.42	2.53	40/1217	37/1464	1.29	0.81–2.04	0.280
c.68-7 T > A	-	3	Not applicable^h^			0.07	0.07	4/2076	1/1458	3.10	0.34–28.31	0.316
c.516 + 18 T > C	-	1	Not applicable^h^			0.29	0.49	8/1207	7/1417	1.41	0.51–3.91	0.511
c.8954-5_8954-2delAACA	-	0	Not applicable^h^			0.06	0.14	2/2088	2/1463	0.83	0.12–5.93	0.855

^a^ Breast Cancer Information Core (http://research.nhgri.nih.gov/bic/), an open access online breast cancer mutation database assessed by Oct 2015

^b^ AGVGD grades: C0, C15, C25, C35, C45, C55, C65 with C65 most likely to interfere with function and C0 least likely. The probability was assessed using the alignment of which from human to sea urchin in *BRCA2*

^c^ PolyPhen-2 grades: Benign, Possibly damaging, Probably damaging

^d^ SIFT grades: Tolerated, Damaging

^e^ Minor allele frequency, which was detected in the entire cohort

^f^ Variant frequency, which was detected only in healthy controls

^g^ Odds ratio, adjusted for ethnicity, age and family history using logistic regression

^h^ AGVGD, PolyPhen-2 and SIFT analyses can only predict the effect of missense variant

In the single-variant association testing using logistic regression, *BRCA1* p.Arg762Ser was associated with breast cancer risk with a marginal significance (OR = 7.4; 95% CI, 0.9–62.3; *p* = 0.06) (Additional file 2: Figure S1). This variant was found in 5 out of 858 Chinese breast cancer patients (0.6%) and 1 out of 1,054 Chinese controls (0.1%) (Additional file 1: Table S3), and was marginally associated with breast cancer risk in Chinese women (OR = 6.7; 95% CI, 0.8–57.6; *p* = 0.08) (Additional file 2: Figure S2). The average age of diagnosis was 39 years old, 25% of women had estrogen receptor (ER) negative breast cancer but none reported any family history of breast or ovarian cancer. The *BRCA1* p.Pro346Ser was associated with breast cancer risk in Chinese women [5 out of 859 Chinese breast cancer patients (0.6%) and 2 out of 1,055 Chinese controls (0.2%), OR = 3.3; 95% CI, 0.6–17.3; *p* = 0.15] (Additional file 1: Table S3a and Additional file 2: Figure S2), but the results were not statistically significant. The average age of diagnosis was 62 years old, 20% of women had ER negative breast cancer but none reported any family history of breast or ovarian cancer. None of the *BRCA2* variants were significantly associated with breast cancer risk either in the overall cohort, or when stratified by ethnicity (Additional file 2: Figure S1 and S2).

The probability that missense variants were deleterious to protein function was assessed by three *in-silico* models, namely AGVGD, PolyPhen-2 and SIFT. Of the 29 missense variants that have been analyzed, three (*BRCA2* p.Ala1996Thr, p.Gly2901Asp and p.Tyr3035Cys) were predicted to be likely pathogenic (Tables 2 and 3).

## Discussion

In this study, we analyzed the frequency of 7 *BRCA1* and 25 *BRCA2* variants from exonic and intronic regions identified previously in Malaysian breast cancer patients by germline analysis. The genotyping of variants was conducted using a high-throughput mass spectrometry platform [18].

The variant frequency suggested that one of the 63 tested variants has a minor allelic frequency of >2% in unaffected women and is likely to be benign [6]. Four variants that had more than 1% of variant frequency in unaffected women could be potentially benign. Of these variants, three (*BRCA2* p.Ile1929Val, p.Lys2729Asn and p.Ile3412Val) have been classified as Class 1 (not pathogenic) and one (*BRCA2* p.Arg2108Cys) has been classified as Class 2 (likely not pathogenic) in either Breast Cancer Information Core Database (http://research.nhgri.nih.gov/bic/) or DatabaseARUP Laboratories *BRCA* Mutation Database (http://arup.utah.edu/database/BRCA/) using different approaches [20, 23]. Moreover, *BRCA2* p.Ile3412Val was also reported by ENIGMA (http://enigmaconsortium.org/) as benign variant to occur in non-founder African control reference group at an allele frequency ≥1%. Although *BRCA2* p.Lys2729Asn was predicted to have damaging effect by PolyPhen-2 and SIFT, the prediction models may have limitation to predict the actual consequences of missense mutation accurately [24] therefore the population frequency supersedes the prediction models [8]. These findings are in accordance with the results of our study which concluded that these variants are benign variants or polymorphisms.

The clinical significance of *BRCA2* p.Cys315Ser and p.Arg2108Cys is currently listed as uncertain, but our study suggests that these variants are likely to be benign. This is consistent with a study in Chinese women from Shanghai in which *BRCA2* p.Cys315Ser was detected in 1.4% of cases and 0.9% of controls, compared with 0.9% of cases and 1.2% of controls in our study [12]. Notably, *BRCA2* p.Arg2108Cys was evaluated as pathogenic in spontaneous homologous recombination [25], but our study suggests that this variant is unlikely to be associated with high risk of breast cancer.

It was estimated that the rare variant had a relative risk of above 2 and above 4 might confer moderate and high risk of breast cancer, respectively [26, 27]. Our study suggests that one *BRCA1* variant may be associated with increased risk of breast cancer. The *BRCA1* p.Arg762Ser may be associated with breast cancer risk with a marginal significance (*p* = 0.06). This variant was previously found in Chinese and Malay women and the clinical significance is currently unknown [12, 28, 29]. Although the *in-silico* analyses predicted the amino acid substitution of this variant is unlikely to have damaging effect to protein function, our study suggests that this warrants further analyses in Asian women.

Notably, three *BRCA2* variants (p.Ala1996Thr, p.Gly2901Asp and p.Tyr3035Cys) which are predicted to be pathogenic by *in-silico* prediction models were found in cases and in healthy controls. The clinical significance of *BRCA2* p.Ala1996Thr is currently listed as uncertain and the substitution of valine at the same codon (p.Ala1996Val) in a Western European woman is also uncertain [7]. *BRCA2* p.Gly2901Asp was suggested as neutral in mouse embryonic stem cell-based functional assay [30], and predicted to be uncertain in protein likelihood ratios [31] and homology-directed repair activity [32]. Although *BRCA2* p.Tyr3035Cys was predicted to be likely deleterious in protein likelihood ratios [31], this variant did not show any significant association with breast cancer risk in our study.

There are several limitations to this study. The breast cancer cases were not age- and ethnicity-matched with controls in this study, but these variables were adjusted for all variant analyses. Another limitation is that majority of the variants selected for this study are rare in our population. These rare variants were detected in very low frequency, thus decrease the statistical power in a case-control study. Analyses in larger groups are necessary to confirm these findings.

## Conclusions

Our data suggests that *BRCA2* p.Ile3412Val is likely to be benign and *BRCA1* p.Arg762Ser is likely to be associated with breast cancer risk. This study could contribute the evidence to support the characterization of variants with uncertain significance in *BRCA1* and *BRCA2*.

## Additional files

Additional file 1: Table S1a.
*BRCA1* variants included in genotyping assay design. A total of 23 *BRCA1* variants were included in the genotyping assay. Of these, two variants were excluded due to genotyping call rate <95%. **Table S1b**. *BRCA2* variants included in genotyping assay design. A total of 44 *BRCA2* variants were included in the genotyping assay. Of these, two variants were excluded due to genotyping call rate <95%. **Table S2**. Characteristics of Malaysian breast cancer cases and healthy controls in ethnicity subgroups: (a) Chinese, (b) Malay and (c) Indian. There was no difference in age for cases and controls for Chinese and Indian women, but healthy women were on average 2 years older than the cases for Malay women. **Table S3a**. Frequency of *BRCA1* variants detected in ethnicity subgroups. The table describes the frequency of *BRCA1* variants detected in Chinese, Malay and Indian women. **Table S3b**. Frequency of *BRCA2* variants detected in ethnicity subgroups. The table describes the frequency of *BRCA2* variants detected in Chinese, Malay and Indian women. (DOCX 195 kb)
Additional file 2: Figure S1. Association of *BRCA1* and *BRCA2* variants with breast cancer risk in all breast cancer cases and healthy controls. The forest plot illustrates the association of *BRCA1* and *BRCA2* variants with breast cancer risk in all breast cancer cases and healthy controls. **Figure S2**. Association of variants with breast cancer risk in ethnicity subgroups: (a) *BRCA1* and (b) *BRCA2*. The forest plot illustrates the association of *BRCA1* and *BRCA2* variants with breast cancer risk in certain ethnicity subgroups that can be analyzed. (DOCX 145 kb)

## Abbreviations

AGVGD: Align-GVGD
CI: Confidence interval
ER: Estrogen receptor
MAF: Minor allele frequency
MLPA: Multiplex ligation-dependent probe amplification
OR: Odds ratio
PolyPhen-2: Polymorphism Phenotyping v2
SIFT: Sorting Tolerant From Intolerant
VUS: Variant of uncertain significance
