# Supplementary material for: Characterization of BRCA1 and BRCA2 variants in multi-ethnic Asian cohort from a Malaysian case-control study
Source: BMC Cancer. 2017 Feb 22;17:149. doi: 10.1186/s12885-017-3099-6 (PMC5320733; doi:10.1186/s12885-017-3099-6)
Supplement: Additional file 1: Table S1a. — BRCA1 variants included in genotyping assay design. A total of 23 BRCA1 variants were included in the genotyping assay. Of these, two variants were excluded due to genotyping call rate <95%. Table S1b. BRCA2 variants included in genotyping assay design. A total of 44 BRCA2 variants were included in the genotyping assay. Of these, two variants were excluded due to genotyping call rate <95%. Table S2. Characteristics of Malaysian breast cancer cases and healthy controls in ethnicity subgroups: (a) Chinese, (b) Malay and (c) Indian. There was no difference in age for cases and controls for Chinese and Indian women, but healthy women were on average 2 years older than the cases for Malay women. Table S3a. Frequency of BRCA1 variants detected in ethnicity subgroups. The table describes the frequency of BRCA1 variants detected in Chinese, Malay and Indian women. Table S3b. Frequency of BRCA2 variants detected in ethnicity subgroups. The table describes the frequency of BRCA2 variants detected in Chinese, Malay and Indian women. (DOCX 195 kb) [file 12885_2017_3099_MOESM1_ESM.docx]

**Table S1a. *BRCA1* variants included in genotyping assay design.**

| **HGVS cDNA** | **HGVS protein** | **Type of variant** | **Source** | **Phase for testing^a^** | **Genotyping call rate (%)** | **Remarks** |
| --- | --- | --- | --- | --- | --- | --- |
| c.200A>G | p.Asp67Gly | Missense | Novel | 2 | 99.74 |  |
| c.571G>A | p.Val191Ile | Missense | BIC, [[1-5](#_ENREF_1)] | 2 | 99.96 |  |
| c.754C>T | p.Arg252Cys | Missense | BIC, [[3](#_ENREF_3)] | 2 | 99.96 |  |
| c.823G>A | p.Gly275Ser | Missense | BIC, [[6](#_ENREF_6), [7](#_ENREF_7)] | 1+2 | 99.94 |  |
| c.1036C>T | p.Pro346Ser | Missense | BIC, [[3](#_ENREF_3), [8](#_ENREF_8)] | 2 | 100.00 |  |
| c.1319T>C | p.Leu440Ser | Missense | BIC | 1+2 | 99.86 |  |
| c.2286A>T | p.Arg762Ser | Missense | BIC, [[3](#_ENREF_3), [5](#_ENREF_5), [9](#_ENREF_9), [10](#_ENREF_10)] | 2 | 99.93 |  |
| c.2519G>A | p.Ser840Asn | Missense | Novel | 2 | 99.22 |  |
| c.2726A>T | p.Asn909Ile | Missense | BIC, [[3](#_ENREF_3), [6](#_ENREF_6)] | 1+2 | 99.07 |  |
| c.2739T>A | p.Asn913Lys | Missense | BIC, [[3](#_ENREF_3)] | 2 | 99.96 |  |
| c.3448C>T | p.Pro1150Ser | Missense | BIC, [[5](#_ENREF_5), [11](#_ENREF_11)] | 2 | 99.37 |  |
| c.3625T>G | p.Leu1209Val | Missense | BIC, [[4](#_ENREF_4)] | 1+2 | 98.99 |  |
| c.3662A>C | p.Glu1221Ala | Missense | BIC, [[3](#_ENREF_3)] | 2 | 99.48 |  |
| c.3803A>G | p.Asn1268Ser | Missense | BIC, [[3](#_ENREF_3)] | 2 | 99.03 |  |
| c.4643C>T | p.Thr1548Met | Missense | BIC | 1+2 | 98.54 |  |
| c.4892G>A | p.Ser1631Asn | Missense | BIC, [[3](#_ENREF_3)] | 1+2 | 98.96 |  |
| c.5057A>G | p.His1686Arg | Missense | [[2](#_ENREF_2)] | 2 | 99.48 |  |
| c.5504G>A | p.Arg1835Gln | Missense | BIC, [[3](#_ENREF_3), [4](#_ENREF_4)] | 2 | 99.96 |  |
| c.-19-3A>G | - | Intronic | BIC, [[10](#_ENREF_10)] | 1+2 | 62.95 | Excluded due to genotyping call rate <95% |
| c.-19-10T>C | - | Intronic | BIC, [[3](#_ENREF_3), [12](#_ENREF_12)] | 1+2 | 83.10 | Excluded due to genotyping call rate <95% |
| c.-19-55_-19-54insT | - | Intronic | BIC | 1+2 | 98.23 |  |
| c.4186-10G>A | - | Intronic | BIC, [[3](#_ENREF_3)] | 1+2 | 99.30 |  |
| c.4675+7T>C | - | Intronic | BIC | 1+2 | 99.94 |  |

^a^ Phase 1 was tested on 879 breast cancer cases; Phase 2 was tested on 1,231 breast cancer cases and 1,493 healthy controls.

**Table S1b. *BRCA2* variants included in genotyping assay design.**

| **HGVS cDNA** | **HGVS protein** | **Type of variant** | **Source** | **Phase for testing^a^** | **Genotyping call rate (%)** | **Remarks** |
| --- | --- | --- | --- | --- | --- | --- |
| c.215A>G | p.Asn72Ser | Missense | BIC, [[3](#_ENREF_3)] | 2 | 99.48 |  |
| c.440A>G | p.Gln147Arg | Missense | BIC, [[3-5](#_ENREF_3), [13](#_ENREF_13)] | 2 | 99.93 |  |
| c.943T>A | p.Cys315Ser | Missense | BIC, [[3](#_ENREF_3), [5](#_ENREF_5), [9](#_ENREF_9), [14](#_ENREF_14), [15](#_ENREF_15), [13](#_ENREF_13)] | 1+2 | 97.72 |  |
| c.1568A>G | p.His523Arg | Missense | [[13](#_ENREF_13)] | 2 | 37.41 | Excluded due to genotyping call rate <95% |
| c.1626A>G | p.Ile542Met | Missense | Novel | 2 | 98.58 |  |
| c.1825C>G | p.Gln609Glu | Missense | BIC, [[3](#_ENREF_3), [4](#_ENREF_4)] | 2 | 99.96 |  |
| c.2186T>C | p.Ile729Thr | Missense | Novel | 2 | 98.25 |  |
| c.2399G>T | p.Gly800Val | Missense | BIC | 1+2 | 99.38 |  |
| c.2678A>G | p.Gln893Arg | Missense | BIC, [[3](#_ENREF_3)] | 2 | 99.81 |  |
| c.2848G>A | p.Val950Ile | Missense | [[4](#_ENREF_4)] | 2 | 99.40 |  |
| c.3445A>G | p.Met1149Val | Missense | BIC, [[3](#_ENREF_3), [4](#_ENREF_4), [9](#_ENREF_9), [13](#_ENREF_13)] | 2 | 99.48 |  |
| c.3782C>G | p.Ser1261Cys | Missense | BIC, [[3](#_ENREF_3)] | 2 | 99.81 |  |
| c.4376A>G | p.Asn1459Ser | Missense | BIC, [[5](#_ENREF_5)] | 1+2 | 99.83 |  |
| c.4779A>C | p.Glu1593Asp | Missense | BIC, [[16](#_ENREF_16)] | 1+2 | 99.92 |  |
| c.5167A>C | p.Thr1723Pro | Missense | BIC, [[3](#_ENREF_3)] | 2 | 99.44 |  |
| c.5312G>A | p.Gly1771Asp | Missense | BIC, [[1](#_ENREF_1), [3](#_ENREF_3), [16](#_ENREF_16)] | 1+2 | 98.82 |  |
| c.5495C>A | p.Ser1832Tyr | Missense | [[17](#_ENREF_17)] | 2 | 99.11 |  |
| c.5624A>C | p.Lys1875Thr | Missense | Novel | 2 | 99.03 |  |
| c.5635G>A | p.Glu1879Lys | Missense | BIC, [[3](#_ENREF_3), [17](#_ENREF_17)] | 2 | 99.48 |  |
| c.5785A>G | p.Ile1929Val | Missense | BIC, [[1](#_ENREF_1), [3](#_ENREF_3), [5](#_ENREF_5), [17](#_ENREF_17)] | 2 | 98.17 |  |
| c.5986G>A | p.Ala1996Thr | Missense | BIC, [[18](#_ENREF_18), [19](#_ENREF_19)] | 1+2 | 99.07 |  |
| c.6322C>T | p.Arg2108Cys | Missense | BIC, [[3](#_ENREF_3), [4](#_ENREF_4), [17](#_ENREF_17), [20](#_ENREF_20)] | 2 | 98.32 |  |
| c.6325G>A | p.Val2109Ile | Missense | BIC, [[5](#_ENREF_5), [15](#_ENREF_15)] | 2 | 98.21 |  |
| c.6826C>A | p.Pro2276Thr | Missense | BIC | 1+2 | 98.42 |  |
| c.6929C>A | p.Thr2310Asn | Missense | BIC, [[3](#_ENREF_3)] | 2 | 99.48 |  |
| c.7052C>G | p.Ala2351Gly | Missense | BIC, [[21](#_ENREF_21), [17](#_ENREF_17)] | 1+2 | 98.40 |  |
| c.7469T>C | p.Ile2490Thr | Missense | BIC, [[5](#_ENREF_5), [17](#_ENREF_17), [14](#_ENREF_14), [22-24](#_ENREF_22)] | 1+2 | 99.07 |  |
| c.7505G>A | p.Arg2502His | Missense | BIC, [[21](#_ENREF_21), [25](#_ENREF_25)] | 2 | 94.34 | Excluded due to genotyping call rate <95% |
| c.8187G>T | p.Lys2729Asn | Missense | BIC, [[1](#_ENREF_1), [3](#_ENREF_3), [5](#_ENREF_5), [26](#_ENREF_26), [14](#_ENREF_14), [15](#_ENREF_15), [23](#_ENREF_23), [24](#_ENREF_24), [27](#_ENREF_27)] | 2 | 99.93 |  |
| c.8356G>A | p.Ala2786Thr | Missense | BIC, [[3](#_ENREF_3), [5](#_ENREF_5)] | 1+2 | 99.47 |  |
| c.8393C>T | p.Pro2798Leu | Missense | BIC | 1+2 | 99.94 |  |
| c.8527A>T | p.Asn2843Tyr | Missense | Novel | 2 | 97.84 |  |
| c.8702G>A | p.Gly2901Asp | Missense | BIC, [[3](#_ENREF_3), [5](#_ENREF_5), [23](#_ENREF_23), [28](#_ENREF_28), [29](#_ENREF_29)] | 2 | 99.96 |  |
| c.9104A>G | p.Tyr3035Cys | Missense | BIC, [[3](#_ENREF_3), [17](#_ENREF_17), [23](#_ENREF_23)] | 2 | 99.44 |  |
| c.9106C>G | p.Gln3036Glu | Missense | BIC, [[3](#_ENREF_3)] | 1+2 | 99.89 |  |
| c.9344A>G | p.Lys3115Arg | Missense | BIC | 1+2 | 99.04 |  |
| c.9538C>T | p.Leu3180Phe | Missense | BIC, [[26](#_ENREF_26)] | 1+2 | 99.49 |  |
| c.9907A>T | p.Ser3303Cys | Missense | BIC, [[3](#_ENREF_3)] | 2 | 99.48 |  |
| c.10234A>G | p.Ile3412Val | Missense | BIC, [[3-5](#_ENREF_3), [17](#_ENREF_17), [26](#_ENREF_26), [14](#_ENREF_14), [16](#_ENREF_16), [30](#_ENREF_30), [13](#_ENREF_13)] | 2 | 99.96 |  |
| c.68-7T>A | - | Intronic | BIC, [[3](#_ENREF_3), [17](#_ENREF_17)] | 1+2 | 99.41 |  |
| c.67+5T>A | - | Intronic | Novel | 1+2 | 99.47 |  |
| c.516+18T>C | - | Intronic | BIC | 2 | 97.84 |  |
| c.7977-23T>C | - | Intronic | Novel | 1+2 | 99.44 |  |
| c.8954-5_8954-2delAACA | - | Intronic | Novel | 1+2 | 99.89 |  |

^a^ Phase 1 was tested on 879 breast cancer cases; Phase 2 was tested on 1,231 breast cancer cases and 1,493 healthy controls.

**Table S2. Characteristics of Malaysian breast cancer cases and healthy controls in ethnicity subgroups: (a) Chinese, (b) Malay and (c) Indian.**

**(a)**

| **Characteristics** | **Breast cancer cases** | | **Healthy controls** | | **p*-*value** |
| --- | --- | --- | --- | --- | --- |
|  | **N=1,394** | | **N=1,071** | |  |
|  | **N** | **%** | **N** | **%** |  |
| Age^a^ (years) | | | | |  |
| Average age | 50.1 |  | 50.4 |  | 0.461 |
| ≤30 | 44 | 3.2 | 0 | 0.0 |  |
| 31-40 | 202 | 14.5 | 54 | 5.0 |  |
| 41-50 | 489 | 35.1 | 549 | 51.3 |  |
| 51-60 | 430 | 30.8 | 366 | 34.2 |  |
| ≥61 | 229 | 16.4 | 102 | 9.5 |  |
| Family history^b^ | | | | | <0.001 |
| Yes | 361 | 25.9 | 205 | 19.1 |  |
| No | 1,029 | 73.8 | 866 | 80.9 |  |
| No data | 4 | 0.3 | 0 | 0.0 |  |
| Pathology profile | | | | |  |
| ER+ | 822 | 59.0 |  |  |  |
| ER- | 275 | 19.7 |  |  |  |
| Triple negative | 150 | 10.8 |  |  |  |
| No data | 147 | 10.5 |  |  |  |

**(b)**

| **Characteristics** | **Breast cancer cases** | | **Healthy controls** | | **p*-*value** |
| --- | --- | --- | --- | --- | --- |
|  | **N=406** | | **N=167** | |  |
|  | **N** | **%** | **N** | **%** |  |
| Age^a^ (years) | | | | |  |
| Average age | 46.2 |  | 48.4 |  | 0.007 |
| ≤30 | 24 | 5.9 | 0 | 0.0 |  |
| 31-40 | 94 | 23.2 | 6 | 3.6 |  |
| 41-50 | 155 | 38.2 | 111 | 66.5 |  |
| 51-60 | 104 | 25.6 | 39 | 23.4 |  |
| ≥61 | 29 | 7.1 | 11 | 6.6 |  |
| Family history^b^ | | | | | 0.838 |
| Yes | 84 | 20.7 | 36 | 21.6 |  |
| No | 320 | 78.8 | 131 | 78.4 |  |
| No data | 2 | 0.5 | 0 | 0.0 |  |
| Pathology profile | | | | |  |
| ER+ | 225 | 55.4 |  |  |  |
| ER- | 99 | 24.4 |  |  |  |
| Triple negative | 56 | 13.8 |  |  |  |
| No data | 26 | 6.4 |  |  |  |

**(c)**

| **Characteristics** | **Breast cancer cases** | | **Healthy controls** | | **p*-*value** |
| --- | --- | --- | --- | --- | --- |
|  | **N=310** | | **N=255** | |  |
|  | **N** | **%** | **N** | **%** |  |
| Age^a^ (years) | | | | |  |
| Average age | 51.1 |  | 51.2 |  | 0.826 |
| ≤30 | 8 | 2.6 | 0 | 0.0 |  |
| 31-40 | 41 | 13.2 | 12 | 4.7 |  |
| 41-50 | 107 | 34.5 | 121 | 47.5 |  |
| 51-60 | 89 | 28.7 | 96 | 37.6 |  |
| ≥61 | 65 | 21.0 | 26 | 10.2 |  |
| Family history^b^ | | | | | 0.708 |
| Yes | 61 | 19.7 | 47 | 18.4 |  |
| No | 249 | 80.3 | 208 | 81.6 |  |
| No data | 0 | 0.0 | 0 | 0.0 |  |
| Pathology profile | | | | |  |
| ER+ | 171 | 55.2 |  |  |  |
| ER- | 77 | 24.8 |  |  |  |
| Triple negative | 36 | 11.6 |  |  |  |
| No data | 26 | 8.4 |  |  |  |

^a^ Age of diagnosis for breast cancer cases or age of consent for healthy controls.

^b^ Family history of breast or ovarian cancer in first or second degree relatives.

**Table S3a. Frequency of *BRCA1* variants detected in ethnicity subgroups.**

| **HGVS cDNA** | **HGVS protein** | **Breast cancer cases** | | | **Healthy controls** | | |
| --- | --- | --- | --- | --- | --- | --- | --- |
|  |  | **Chinese** | **Malay** | **Indian** | **Chinese** | **Malay** | **Indian** |
| c.571G>A | p.Val191Ile | 4/858 | 1/221 | 0/139 | 9/1055 | 1/167 | 1/242 |
| c.823G>A | p.Gly275Ser | 0/1385 | 0/397 | 4/307 | 0/1055 | 0/167 | 2/242 |
| c.1036C>T | p.Pro346Ser | 5/859 | 0/221 | 0/139 | 2/1055 | 0/167 | 0/242 |
| c.2286A>T | p.Arg762Ser | 5/858 | 1/221 | 0/139 | 1/1054 | 0/167 | 0/242 |
| c.2726A>T | p.Asn909Ile | 5/1386 | 0/398 | 0/307 | 4/1053 | 0/167 | 0/242 |
| c.3625T>G | p.Leu1209Val | 0/1362 | 1/394 | 0/302 | 0/1052 | 2/167 | 0/242 |
| c.3662A>C | p.Glu1221Ala | 1/858 | 0/220 | 0/137 | 3/1045 | 0/167 | 0/242 |

**Table S3b. Frequency of *BRCA2* variants detected in ethnicity subgroups.**

| **HGVS cDNA** | **HGVS protein** | **Breast cancer cases** | | | **Healthy controls** | | |
| --- | --- | --- | --- | --- | --- | --- | --- |
|  |  | **Chinese** | **Malay** | **Indian** | **Chinese** | **Malay** | **Indian** |
| c.215A>G | p.Asn72Ser | 1/858 | 0/220 | 0/137 | 3/1045 | 0/167 | 0/242 |
| c.440A>G | p.Gln147Arg | 7/858 | 5/221 | 0/139 | 5/1054 | 4/167 | 0/242 |
| c.943T>A | p.Cys315Ser | 18/1355 | 0/393 | 0/307 | 17/1045 | 0/155 | 0/220 |
| c.1825C>G | p.Gln609Glu | 0/858 | 2/221 | 0/139 | 0/1055 | 1/167 | 0/242 |
| c.2186T>C | p.Ile729Thr | 1/854 | 0/220 | 0/139 | 4/1047 | 0/155 | 0/221 |
| c.3445A>G | p.Met1149Val | 0/858 | 7/220 | 0/137 | 3/1045 | 4/167 | 0/242 |
| c.4376A>G | p.Asn1459Ser | 5/1385 | 0/396 | 0/306 | 2/1054 | 0/167 | 0/241 |
| c.4779A>C | p.Glu1593Asp | 0/1386 | 1/397 | 5/305 | 0/1055 | 1/167 | 3/242 |
| c.5167A>C | p.Thr1723Pro | 0/858 | 1/219 | 0/137 | 1/1045 | 0/167 | 0/242 |
| c.5312G>A | p.Gly1771Asp | 0/1360 | 1/393 | 0/301 | 1/1051 | 0/167 | 0/241 |
| c.5785A>G | p.Ile1929Val | 18/854 | 0/220 | 2/139 | 23/1045 | 0/155 | 0/221 |
| c.5986G>A | p.Ala1996Thr | 0/1364 | 0/394 | 6/302 | 0/1053 | 0/167 | 5/242 |
| c.6322C>T | p.Arg2108Cys | 9/850 | 4/220 | 0/137 | 15/1040 | 1/158 | 0/233 |
| c.6325G>A | p.Val2109Ile | 4/854 | 2/220 | 0/139 | 6/1046 | 1/155 | 0/221 |
| c.7052C>G | p.Ala2351Gly | 7/1377 | 1/393 | 0/307 | 5/1046 | 2/155 | 0/221 |
| c.7469T>C | p.Ile2490Thr | 3/1364 | 0/394 | 0/302 | 1/1053 | 0/167 | 0/242 |
| c.8187G>T | p.Lys2729Asn | 15/858 | 0/221 | 0/139 | 18/1054 | 1/167 | 0/242 |
| c.8356G>A | p.Ala2786Thr | 7/1384 | 1/397 | 0/307 | 4/1048 | 0/167 | 0/233 |
| c.8702G>A | p.Gly2901Asp | 1/858 | 0/221 | 0/138 | 5/1055 | 0/167 | 0/242 |
| c.9104A>G | p.Tyr3035Cys | 2/858 | 0/219 | 0/136 | 1/1045 | 0/167 | 0/242 |
| c.9538C>T | p.Leu3180Phe | 0/1385 | 1/397 | 0/307 | 2/1048 | 0/167 | 0/233 |
| c.10234A>G | p.Ile3412Val | 34/858 | 3/221 | 3/138 | 35/1055 | 2/167 | 0/242 |
| c.68-7T>A | - | 1/1377 | 1/396 | 2/303 | 0/1053 | 0/167 | 1/238 |
| c.516+18T>C | - | 8/850 | 0/220 | 0/137 | 7/1049 | 0/152 | 0/216 |
| c.8954-5_8954-2delAACA | - | 2/1383 | 0/398 | 0/307 | 2/1054 | 0/167 | 0/242 |

**Supplementary Data Reference**

1. Easton DF, Deffenbaugh AM, Pruss D, Frye C, Wenstrup RJ, Allen-Brady K et al. A systematic genetic assessment of 1,433 sequence variants of unknown clinical significance in the BRCA1 and BRCA2 breast cancer-predisposition genes. American journal of human genetics. 2007;81(5):873-83. doi:10.1086/521032.

2. Bouwman P, van der Gulden H, van der Heijden I, Drost R, Klijn CN, Prasetyanti P et al. A high-throughput functional complementation assay for classification of BRCA1 missense variants. Cancer discovery. 2013;3(10):1142-55. doi:10.1158/2159-8290.CD-13-0094.

3. Thirthagiri E, Lee SY, Kang P, Lee DS, Toh GT, Selamat S et al. Evaluation of BRCA1 and BRCA2 mutations and risk-prediction models in a typical Asian country (Malaysia) with a relatively low incidence of breast cancer. Breast cancer research : BCR. 2008;10(4):R59. doi:10.1186/bcr2118.

4. Purnomosari D, Pals G, Wahyono A, Aryandono T, Manuaba TW, Haryono SJ et al. BRCA1 and BRCA2 germline mutation analysis in the Indonesian population. Breast cancer research and treatment. 2007;106(2):297-304. doi:10.1007/s10549-006-9493-4.

5. Suter NM, Ray RM, Hu YW, Lin MG, Porter P, Gao DL et al. BRCA1 and BRCA2 mutations in women from Shanghai China. Cancer epidemiology, biomarkers & prevention : a publication of the American Association for Cancer Research, cosponsored by the American Society of Preventive Oncology. 2004;13(2):181-9.

6. Burk-Herrick A, Scally M, Amrine-Madsen H, Stanhope MJ, Springer MS. Natural selection and mammalian BRCA1 sequences: elucidating functionally important sites relevant to breast cancer susceptibility in humans. Mammalian genome : official journal of the International Mammalian Genome Society. 2006;17(3):257-70. doi:10.1007/s00335-005-0067-2.

7. Rajasekaran R, Sudandiradoss C, Doss CG, Sethumadhavan R. Identification and in silico analysis of functional SNPs of the BRCA1 gene. Genomics. 2007;90(4):447-52. doi:10.1016/j.ygeno.2007.07.004.

8. Kuo WH, Lin PH, Huang AC, Chien YH, Liu TP, Lu YS et al. Multimodel assessment of BRCA1 mutations in Taiwanese (ethnic Chinese) women with early-onset, bilateral or familial breast cancer. Journal of human genetics. 2012;57(2):130-8. doi:10.1038/jhg.2011.142.

9. Toh GT, Kang P, Lee SS, Lee DS, Lee SY, Selamat S et al. BRCA1 and BRCA2 germline mutations in Malaysian women with early-onset breast cancer without a family history. PloS one. 2008;3(4):e2024. doi:10.1371/journal.pone.0002024.

10. Cao W, Wang X, Gao Y, Yang H, Li JC. BRCA1 germ-line mutations and tumor characteristics in eastern Chinese women with familial breast cancer. Anatomical record. 2013;296(2):273-8. doi:10.1002/ar.22628.

11. Abkevich V, Zharkikh A, Deffenbaugh AM, Frank D, Chen Y, Shattuck D et al. Analysis of missense variation in human BRCA1 in the context of interspecific sequence variation. Journal of medical genetics. 2004;41(7):492-507.

12. Fetzer S, Tworek HA, Piver MS, DiCioccio RA. Classification of IVS1-10T-->C as a polymorphism of BRCA1. Cancer genetics and cytogenetics. 1999;113(1):58-64.

13. Zhong L, Zhu ZZ, Shen Y, Sun G, Zhao X, Zhang S et al. Frequent germline mutation in the BRCA2 gene in esophageal squamous cell carcinoma patients from a low-risk Chinese population. Asian Pacific journal of cancer prevention : APJCP. 2011;12(7):1771-6.

14. Akbari MR, Malekzadeh R, Nasrollahzadeh D, Amanian D, Islami F, Li S et al. Germline BRCA2 mutations and the risk of esophageal squamous cell carcinoma. Oncogene. 2008;27(9):1290-6. doi:10.1038/sj.onc.1210739.

15. Hu N, Wang C, Han XY, He LJ, Tang ZZ, Giffen C et al. Evaluation of BRCA2 in the genetic susceptibility of familial esophageal cancer. Oncogene. 2004;23(3):852-8. doi:10.1038/sj.onc.1207150.

16. Wagner TM, Hirtenlehner K, Shen P, Moeslinger R, Muhr D, Fleischmann E et al. Global sequence diversity of BRCA2: analysis of 71 breast cancer families and 95 control individuals of worldwide populations. Human molecular genetics. 1999;8(3):413-23.

17. Borg A, Haile RW, Malone KE, Capanu M, Diep A, Torngren T et al. Characterization of BRCA1 and BRCA2 deleterious mutations and variants of unknown clinical significance in unilateral and bilateral breast cancer: the WECARE study. Human mutation. 2010;31(3):E1200-40. doi:10.1002/humu.21202.

18. Kanchi KL, Johnson KJ, Lu C, McLellan MD, Leiserson MD, Wendl MC et al. Integrated analysis of germline and somatic variants in ovarian cancer. Nature communications. 2014;5:3156. doi:10.1038/ncomms4156.

19. Levanat S, Musani V, Cvok ML, Susac I, Sabol M, Ozretic P et al. Three novel BRCA1/BRCA2 mutations in breast/ovarian cancer families in Croatia. Gene. 2012;498(2):169-76. doi:10.1016/j.gene.2012.02.010.

20. Balia C, Galli A, Caligo MA. Effect of the overexpression of BRCA2 unclassified missense variants on spontaneous homologous recombination in human cells. Breast cancer research and treatment. 2011;129(3):1001-9. doi:10.1007/s10549-011-1607-y.

21. Spearman AD, Sweet K, Zhou XP, McLennan J, Couch FJ, Toland AE. Clinically applicable models to characterize BRCA1 and BRCA2 variants of uncertain significance. Journal of clinical oncology : official journal of the American Society of Clinical Oncology. 2008;26(33):5393-400. doi:10.1200/JCO.2008.17.8228.

22. Freedman ML, Penney KL, Stram DO, Le Marchand L, Hirschhorn JN, Kolonel LN et al. Common variation in BRCA2 and breast cancer risk: a haplotype-based analysis in the Multiethnic Cohort. Human molecular genetics. 2004;13(20):2431-41. doi:10.1093/hmg/ddh270.

23. Karchin R, Agarwal M, Sali A, Couch F, Beattie MS. Classifying Variants of Undetermined Significance in BRCA2 with protein likelihood ratios. Cancer informatics. 2008;6:203-16.

24. Biswas K, Das R, Alter BP, Kuznetsov SG, Stauffer S, North SL et al. A comprehensive functional characterization of BRCA2 variants associated with Fanconi anemia using mouse ES cell-based assay. Blood. 2011;118(9):2430-42. doi:10.1182/blood-2010-12-324541.

25. Gayther SA, Russell P, Harrington P, Antoniou AC, Easton DF, Ponder BA. The contribution of germline BRCA1 and BRCA2 mutations to familial ovarian cancer: no evidence for other ovarian cancer-susceptibility genes. American journal of human genetics. 1999;65(4):1021-9. doi:10.1086/302583.

26. Chan M, Ji SM, Yeo ZX, Gan L, Yap E, Yap YS et al. Development of a next-generation sequencing method for BRCA mutation screening: a comparison between a high-throughput and a benchtop platform. The Journal of molecular diagnostics : JMD. 2012;14(6):602-12. doi:10.1016/j.jmoldx.2012.06.003.

27. Farrugia DJ, Agarwal MK, Pankratz VS, Deffenbaugh AM, Pruss D, Frye C et al. Functional assays for classification of BRCA2 variants of uncertain significance. Cancer research. 2008;68(9):3523-31. doi:10.1158/0008-5472.CAN-07-1587.

28. Kuznetsov SG, Liu P, Sharan SK. Mouse embryonic stem cell-based functional assay to evaluate mutations in BRCA2. Nature medicine. 2008;14(8):875-81. doi:10.1038/nm.1719.

29. Guidugli L, Pankratz VS, Singh N, Thompson J, Erding CA, Engel C et al. A classification model for BRCA2 DNA binding domain missense variants based on homology-directed repair activity. Cancer research. 2013;73(1):265-75. doi:10.1158/0008-5472.CAN-12-2081.

30. Kaushal M, Chattopadhyay I, Phukan R, Purkayastha J, Mahanta J, Kapur S et al. Contribution of germ line BRCA2 sequence alterations to risk of familial esophageal cancer in a high-risk area of India. Diseases of the esophagus : official journal of the International Society for Diseases of the Esophagus / ISDE. 2010;23(1):71-5. doi:10.1111/j.1442-2050.2009.00975.x.
