# Supplementary material for: Characterization of BRCA1 and BRCA2 variants in multi-ethnic Asian cohort from a Malaysian case-control study
Source: BMC Cancer. 2017 Feb 22;17:149. doi: 10.1186/s12885-017-3099-6 (PMC5320733; doi:10.1186/s12885-017-3099-6)
Supplement: Additional file 2: Figure S1. — Association of BRCA1 and BRCA2 variants with breast cancer risk in all breast cancer cases and healthy controls. The forest plot illustrates the association of BRCA1 and BRCA2 variants with breast cancer risk in all breast cancer cases and healthy controls. Figure S2. Association of variants with breast cancer risk in ethnicity subgroups: (a) BRCA1 and (b) BRCA2. The forest plot illustrates the association of BRCA1 and BRCA2 variants with breast cancer risk in certain ethnicity subgroups that can be analyzed. (DOCX 145 kb) [file 12885_2017_3099_MOESM2_ESM.docx]

**Figure S1. Association of *BRCA1* and *BRCA2* variants with breast cancer risk in all breast cancer cases and healthy controls.**

**
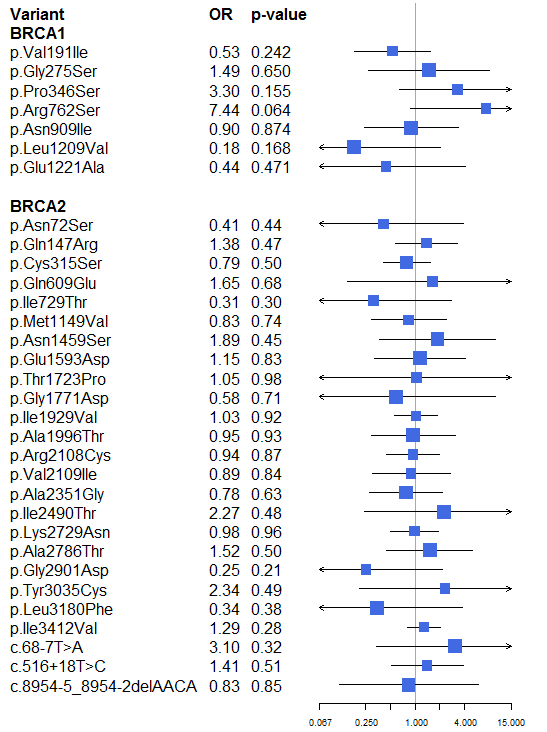
**

**Figure S2. Association of variants with breast cancer risk in ethnicity subgroups: (a) *BRCA1* and (b) *BRCA2*.**

**(a) (b)**

**
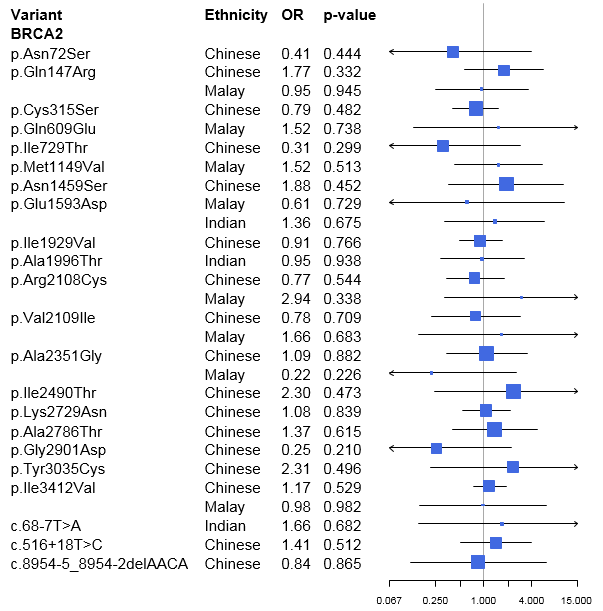

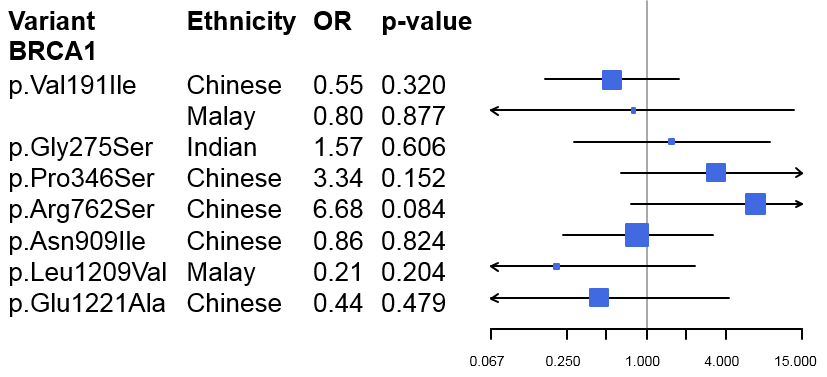
**

Note: Some variants were only present in certain ethnicity. Only those variants that were present in both breast cancer cases and healthy controls among the same ethnicity were analyzed for association with breast cancer risk.
